# Experimental Study on Slicing Sapphire Crystal with Ultrasonic-Assisted Diamond Wire Saw

**DOI:** 10.3390/mi17070867

**Published:** 2026-07-22

**Authors:** Faroug Ismael, Pengfei Sun, Yihe Liu, Honghao Li, Yufei Gao

**Affiliations:** 1 Key Laboratory of High Efficiency and Clean Mechanical Manufacture of Ministry of Education, School of Mechanical Engineering, Shandong University, Jinan 250061, China; 2 Department of Mechanical Engineering, College of Engineering Science, Nyala University, Nyala 63311, Sudan; 3 State Key Laboratory of Advanced Equipment and Technology for Metal Forming, Shandong University, Jinan 250061, China

**Keywords:** sapphire, ultrasonic-assisted diamond wire sawing, surface roughness, surface waviness, orthogonal experiment, regression modeling

## Abstract

Sapphire crystal, owing to its high hardness, chemical inertness, thermal stability, optical transparency, and superior dielectric strength, as well as resistance to scratching, abrasion, friction, and wear, is widely utilized in a broad range of engineering applications. Slicing is the most critical step in sapphire industry processing, as it largely dictates the final surface quality and morphology. Conventional wire sawing methods often lead to undesirable surface defects, while ultrasonic-assisted diamond wire sawing (UADWS) offers potential advantages through enhanced abrasive self-sharpening, micro-hammering, and improved lubricant penetration. However, its influence on sapphire slicing remains insufficiently studied. This study investigates the effects of UADWS parameters—ultrasonic amplitude, horn application position, feed speed, and wire speed—on the surface quality of sapphire crystals. Both single-factor and orthogonal five-level experiments were designed, taking wire and feed speed within industrial parameter ranges. Surface roughness (*Ra*) and waviness peak–valley (PV) difference were used as evaluation indices, and range and variance analyses were performed. In addition, power regression models were developed to predict Ra and PV under varying conditions. The surface morphology results from single-factor experiments reveal that increasing feed speed and wire speed reduces the effectiveness of ultrasonic assistance, while application horn position exerts only a minor influence. Overall, orthogonal analysis confirmed that the relative influence of process parameters on surface quality follows the order: feed speed > wire speed > amplitude > application horn position. These findings establish a foundation for optimizing the sawing and ultrasonic parameters of UADWS to enhance sapphire surface quality, reduce downstream processing requirements, and clarify the importance of controlling feed speed and wire speed.

## 1. Introduction

Sapphire, owing to its exceptional combination of physical, mechanical, thermal, electrical, and optical properties—including high hardness, chemical inertness, thermal stability, optical transparency, and superior dielectric strength, as well as resistance to scratching, abrasion, friction, and wear—is widely utilized in a broad range of engineering applications [1,2,3]. These characteristics make sapphire a critical material in several industries, including extensive use as a substrate in optoelectronic and semiconductor devices [4,5], biomedical applications such as surgical instruments and wearable devices [6], and abrasion-resistant windows for military and industrial purposes [7,8]. The surface properties of sapphire wafers are key indicators of cutting quality, as cracks, pits, and roughness generated during slicing directly influence subsequent grinding and polishing processes. Since slicing is the most critical step in sapphire processing, it largely dictates the final surface quality, integrity, and morphology. Producing a defect-free, smooth surface remains highly challenging due to sapphire’s high hardness and low fracture toughness, which make machining difficult [9]. Multiple machining methods—including thermal, chemical, and mechanical processes—have been employed, each with advantages but also significant constraints. Thermal machining, such as laser processing [10,11,12,13,14], removes sapphire through localized melting or vaporization but often causes undesirable oxidation and heat-affected zones (HAZs) [15,16,17,18], leading to residual stresses and microcracks. Chemical machining employs chemical etchants for precise material removal while minimizing thermal damage; however, it is hindered by slow processing, undercutting, high chemical disposal costs, and limitations in fabricating deep or complex features [19,20,21]. Mechanical machining primarily relies on abrasive techniques, removing material via micro-fracturing, but often results in high cutting forces, friction, microcracking, subsurface damage, and irregular removal, reducing wafer precision and surface quality [22,23]. Conventional outer diameter sawing, once common, is limited by high material loss and low productivity, though improvements such as horizontal oscillation enhanced its performance [24]. The inner diameter or annular saw offers better wafer quality and yields but suffers from greater kerf loss and slower operation compared to newer methods [25]. Free abrasive wire sawing (FAWS) has also been explored; however, only a few studies have addressed sapphire slicing, which is complicated by its hardness and pronounced crystal anisotropy [26,27,28,29]. Fixed diamond abrasive wire sawing (DWS) has emerged as a more efficient alternative, particularly for high-cost, hard materials. This technique employs diamond-impregnated wires to improve cutting efficiency, minimize kerf loss, and enhance surface quality. Its reduced cutting depth also promotes ductile cutting modes, thereby improving surface integrity and morphology in sapphire machining [30,31,32,33].

Extensive research has examined how DWS process parameters influence wafer surface characteristics, particularly morphology and roughness. Wire speed, feed rate, wire tension, and abrasive size significantly affect surface quality [34,35,36]. Cheng et al. [37] and Gupta et al. [38] reported that higher wire speeds reduce surface roughness, while Costa et al. [39] found that increasing wire speed promotes ductile material removal and lower roughness, whereas higher feed rate and wire tension favor brittle removal, increasing roughness. Similar results were reported for mono-Si by Wang et al. [40]. Guo et al. [41,42] showed that lowering the feed rate and increasing the wire speed reduces cutting force and improves surface quality, while Yeh et al. [43] observed better roughness with smaller abrasives during sapphire cutting. Zhu et al. [44] reported that increasing the speed ratio deteriorates the morphology of as-sawn sapphire wafers, raising roughness. Typical surface roughness values remain below 2 μm. However, adjusting only the controllable parameter process of feed and wire speed is insufficient to reduce or mitigate pits, micro-cracks, and morphological irregularities. Cutting fluids aid stable cutting through cooling and lubrication [45,46,47,48], but in DWS, their penetration into the cutting zone is limited, reducing stability and surface integrity. To address these issues, mechanical-assisted methods such as ultrasonic-assisted diamond wire sawing (UADWS) have been introduced for hard and brittle materials [49,50,51,52,53]. Ultrasonic assistance improves material removal and lubricant penetration, yielding more stable cutting and enhanced surface quality, integrity, and morphology. Reported studies show that UADWS surfaces exhibit a mixed texture dominated by pits, micropits, and punctate pits, with roughness generally decreasing at higher amplitudes. Li et al. [54] found that UADWS reduced Ra by up to 55% compared with DWS, with higher wire speeds further lowering roughness. Yan et al. [51] observed minimized pit size in composite ceramics, while Wang et al. [55] and Li et al. [56] demonstrated that UADWS produced smaller, more numerous pits and lower roughness than DWS in mono-Si and SiC. Li et al. [57] reported similar findings with a more punctate pit density and a smaller depth. Furthermore, they noted that amplitude negatively correlates with roughness, whereas feed rate and horn position correlate positively.

In addition to roughness, DWS commonly generates surface waviness, appearing as periodic undulations along the feed direction. This larger-scale deviation, characterized by alternating peaks and valleys, is quantified by the peak–valley (PV) value [58]. While both roughness and waviness use arithmetic mean deviations, PV represents larger geometric deviations and more accurately tracks how periodic waviness evolves under different cutting conditions. Such undulations degrade surface quality, increase downstream polishing costs, and induce stress concentrations, weakening structural integrity and raising fracture risk in as-sawn wafers [59,60]. Waviness is strongly affected by cutting parameters: insufficient forward force causes local oscillations, increasing PV [61], and higher feed rates further elevate PV [62]. Periodic saw marks in sapphire and NdFeB have been linked to reciprocating wire motion and guide wheel misalignment [58,63], while bowing and lateral wire swing also contribute [31]. Mitigation strategies include reciprocating movement-assisted DWS (RMA-DWS), which reduced PV in Si_3_N_4_ [64], and UADWS, which has significantly decreased PV amplitudes in SiC and NdFeB due to improved micro-grinding and intermittent contact [54,57].

Although UADWS has demonstrated clear potential to improve both roughness and waviness PV, no comprehensive study has systematically examined its parameter effects on sapphire slicing. Studying Ra and PV in relation to process factors such as wire speed, feed rate, amplitude, and horn position is therefore essential to improve surface quality and reduce manufacturing costs.

In summary, the application of UADWS to sapphire cutting has not yet been reported, and research on the surface quality of sapphire processed with this technique remains lacking. To address this gap, this work conducts both single-factor and orthogonal experiments on sapphire slicing within industrial parameter ranges under UADWS. Surface properties are evaluated in terms of morphology, roughness, and PV. Furthermore, the effects of amplitude, horn application position, feed rate, and wire speed on sapphire surfaces are analyzed, and regression models are developed based on experimental data. The findings provide a foundation for optimizing UADWS parameters to enhance surface quality in sapphire machining.

## 2. Materials and Methods

### 2.1. Experimental Materials and Equipment

**Figure 1:**
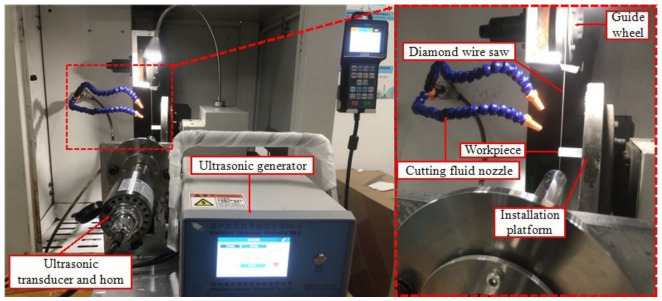
Experimental equipment and apparatus.

**Figure 2:**
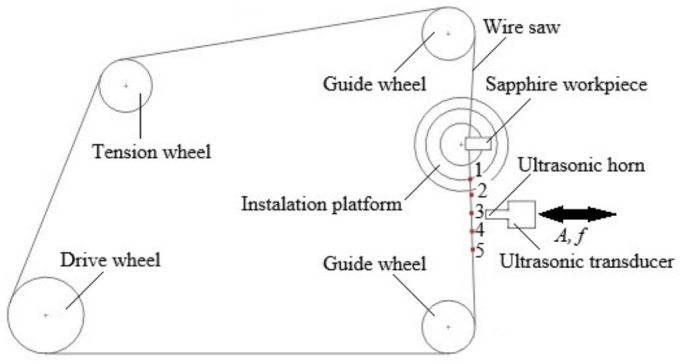
Schematic diagram of the wire saw ultrasonic-assisted cutting principle. Numbers indicate the horn positions from 10 to 50 mm, and the double-headed arrows indicate the ultrasonic vibration direction.

**Figure 3:**
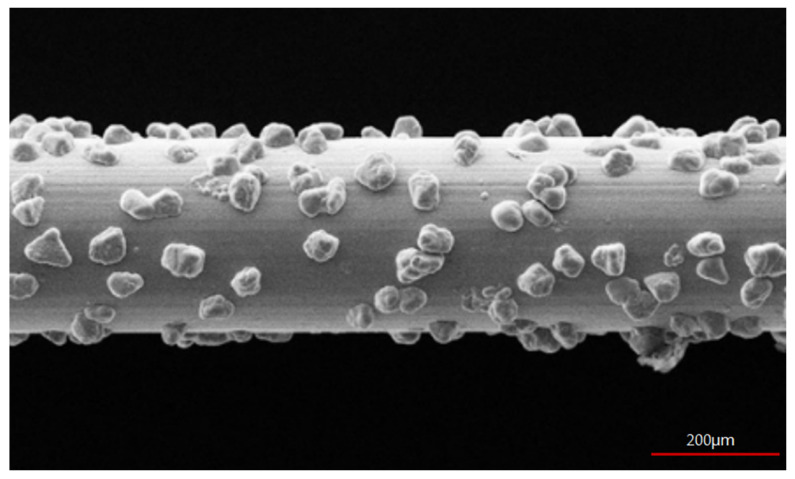
Diamond wire appearance.

The sawing experiments were conducted using an SH300 reciprocating diamond wire sawing machine (Guangzhou Shenghai Electronic Technology Co., Ltd., Guangzhou, China). An SGX ball-screw linear translation module was installed on the worktable to provide precise and stable workpiece feed motion. The diamond wire, supplied by Shantian New Materials Co., Ltd. (Linyi, China), was driven by a drive wheel and guided by two guide wheels, while a gravity-based tensioning system maintained a constant wire tension. The technical specifications of the diamond wire are listed in Table 1. Ultrasonic-assisted cutting was implemented using an ultrasonic vibration system. High-frequency vibration was transmitted to the diamond wire through an ultrasonic horn in a direction perpendicular to the wire motion, thereby assisting material removal during sawing. Figure 1 shows the experimental apparatus, Figure 2 illustrates the ultrasonic–assisted cutting principle, and Figure 3 shows the wire-saw appearance.

At the beginning of each experiment, a rectangular sapphire workpiece supplied by Ningbo Xinlin Magnetic Industry Co., Ltd. (Ningbo, China) was securely fixed to the motorized platform. The workpiece remained mounted while the diamond wire successively sliced wafers with a thickness of 1 mm. Tap water was continuously supplied as the coolant and lubricant to reduce heat and friction, remove cutting debris, maintain stable cutting conditions, and extend the service life of the diamond wire.

Table 1 lists the technical details of the saw wire, ultrasonic system, and specimen sizes. This setup ensures controlled cutting and reliable results.

### 2.2. Experimental Scheme

#### 2.2.1. Single-Factor Experimental Design

To investigate the effects of ultrasonic amplitude A, diamond wire speed $V_{s}$, feed speed $V_{f}$, and horn application position L on the cutting characteristics of sapphire during ultrasonic-assisted diamond wire sawing, four sets of single-factor experiments were designed. The specific experimental parameters are listed in Table 2. Because the wear condition of the diamond wire significantly affects cutting performance and the resulting wafer surface quality [65,66], all cutting trials were conducted under a stabilized wire-wear condition. For each set of process parameters, three slices were cut consecutively using the same wire, after which the wire was replaced before testing the next parameter condition. This procedure limited cumulative wire wear and maintained comparable cutting conditions among the experimental groups, thereby minimizing the influence of wire wear on the experimental results.

The wire-speed and feed-speed ranges were selected to represent conditions commonly used in industrial production. In the single-factor experiments, the waviness peak-to-valley value, PV, was adopted as the evaluation index because the waviness of saw marks has a greater influence on subsequent grinding and polishing processes than surface roughness.

In Single-Factor Experiment 1, $V_{s}$, $V_{f}$, and L were kept constant, while A was varied to investigate its effect on PV. In Single-Factor Experiment 2, A, $V_{f}$, and L were held constant, while $V_{s}$ was varied to evaluate the effect of diamond wire speed on PV. In Single-Factor Experiment 3, A, $V_{s}$, and L were maintained constant, while $V_{f}$ was varied to determine the influence of feed speed on PV. In Single-Factor Experiment 4, A, $V_{s}$, and $V_{f}$ were kept constant, while L was varied to assess the effect of horn application position on PV.

#### 2.2.2. Orthogonal Experimental Design

To further explore the degree of influence of amplitude (A), horn application position (L), feed speed $\left(V_{f}\right)$, and diamond wire speed $\left(V_{s}\right)$ on the cutting characteristics, a four-factor, five-level orthogonal experiment was designed. Each factor was set at five levels, with specific factors, levels, and parameter combinations detailed in Table 3 and Table 4. Surface roughness (Ra) and waviness PV value (peak–valley difference) were used as evaluation criteria. Minitab Statistical Software 2020 (Minitab, LLC, State College, PA, USA) was used for range and mean level analysis.

The degree of influence of each factor was systematically analyzed. Additionally, based on the experimental data, corresponding mathematical models were fitted, which can effectively guide the prediction of surface quality under different process parameter conditions in practical production.

### 2.3. Evaluation Method of Sawing Performance

The reciprocating motion of the diamond wire induces periodic lateral oscillations, resulting in ripple-like saw marks on the sliced surface. Surface roughness and waviness are commonly used to quantitatively characterize the surface quality of diamond wire-sawn wafers [56,57]. In this study, surface waviness was evaluated using the peak-to-valley (PV) value, whereas surface roughness was evaluated using the arithmetic mean roughness (*Ra*). All measurements were performed along the feed direction of the diamond wire.

For each set of process parameters, three slices were produced. Before measurement, each slice was ultrasonically cleaned to remove residual particles and contaminants that could affect the surface characterization. Surface profiles were measured using a KEYENCE laser confocal microscope (VK-X200K, Keyence Corporation, Osaka, Japan). Five randomly selected locations on each slice were measured for both PV and *Ra*. Thus, a total of 15 measurements were obtained for each parameter combination, and their average was used as the representative PV or *Ra* value for that condition. This procedure improved the reliability of the results and minimized the influence of random measurement errors. The resulting PV and *Ra* values were then used to evaluate the effects of ultrasonic amplitude, horn application position, feed speed, and diamond wire speed on the surface quality of the sapphire slices.

## 3. Results

### 3.1. Analysis of Single-Factor Experimental Results

#### 3.1.1. Influence of Amplitude on Surface Waviness PV

**Figure 4:**
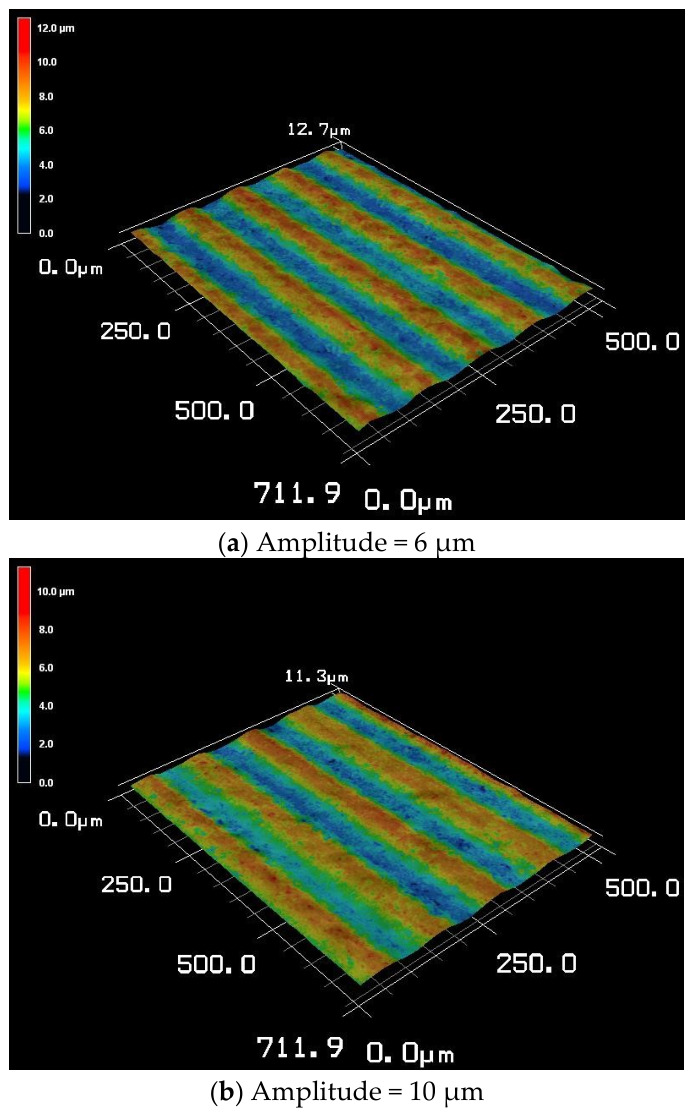

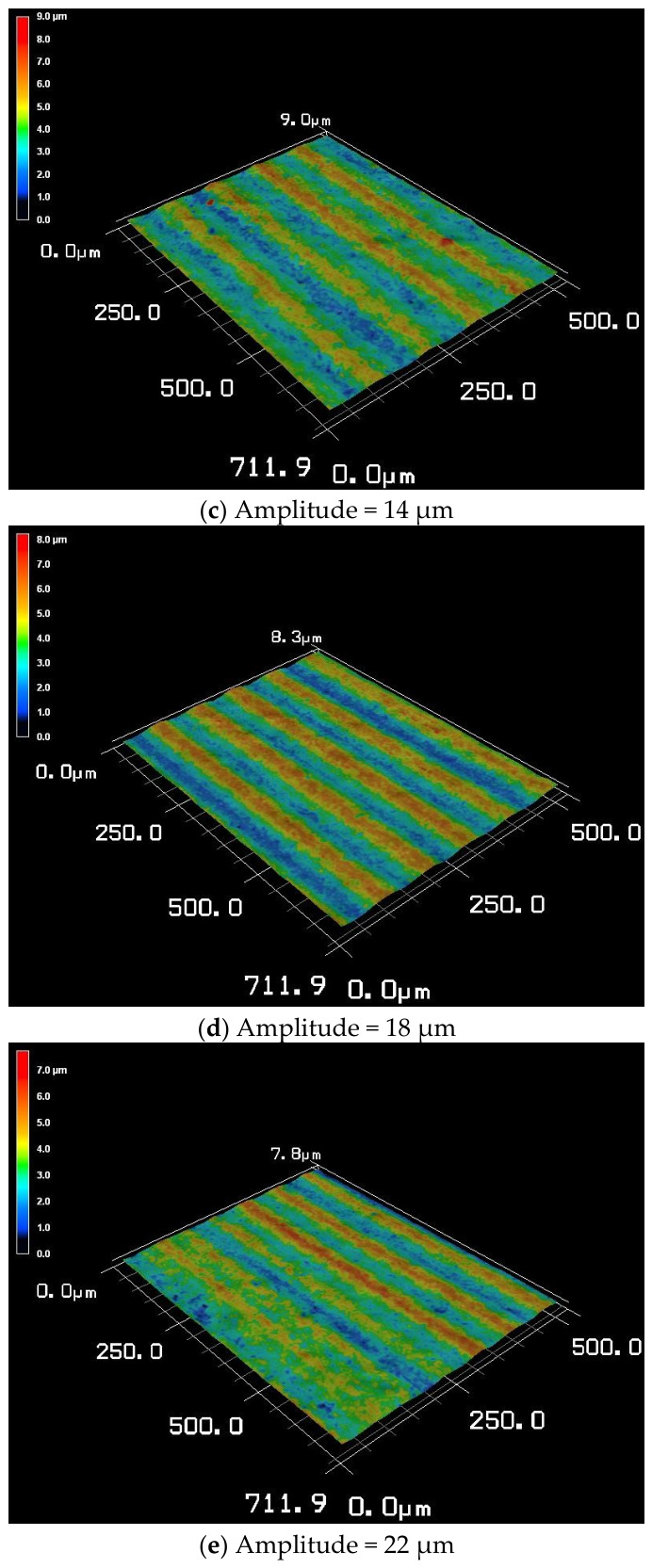
Waviness PV morphology of as-sawn sapphire surface at various amplitudes (application position = 30 mm, $V_{f}$ = 0.3 mm/min, $V_{s}$ = 1200 m/min). (**a**) Amplitude = 6 µm. (**b**) Amplitude = 10 µm. (**c**) Amplitude = 14 µm. (**d**) Amplitude = 18 µm. (**e**) Amplitude = 22 µm.

To more intuitively illustrate the changes in surface waviness PV, a wider range of three-dimensional images of the sliced surfaces was analyzed. Figure 4. presents the 3D surface morphology obtained under a wire speed of 1200 m·min^−1^, a feed speed of 0.3 mm·min^−1^, and a horn application position of 30 mm on the wire saw, where the waviness PV is clearly visible. It can be observed that as the amplitude increases, the waviness PV exhibits a decreasing trend.

This behavior can be attributed to the nature of ultrasonic-assisted wire sawing. When the wire saw is positioned on one side of the kerf during cutting, ultrasonic vibration superimposes high-frequency and varying-amplitude oscillations onto the conventional cutting motion, causing the wire to oscillate slightly in the vertical direction while moving axially. This high-frequency vertical motion with increasing amplitude facilitates continuous self-sharpening of the abrasives bonded to the wire matrix, enhances material removal efficiency, and significantly reduces cutting resistance. Consequently, the cutting process becomes more stable with lower forward forces, which suppresses localized oscillations that typically lead to deeper surface waviness PV. Simultaneously, ultrasonic vibration exerts a minor grinding effect on the peaks and valleys of the emerging wire marks, effectively trimming their tips. As a result, the waviness PV is progressively reduced, mitigating the presence of pronounced wire marks on the slice surface, as clearly demonstrated in Figure 4e when the amplitude reaches 22 μm.

#### 3.1.2. Influence of Wire Speed on Surface Waviness PV

**Figure 5:**
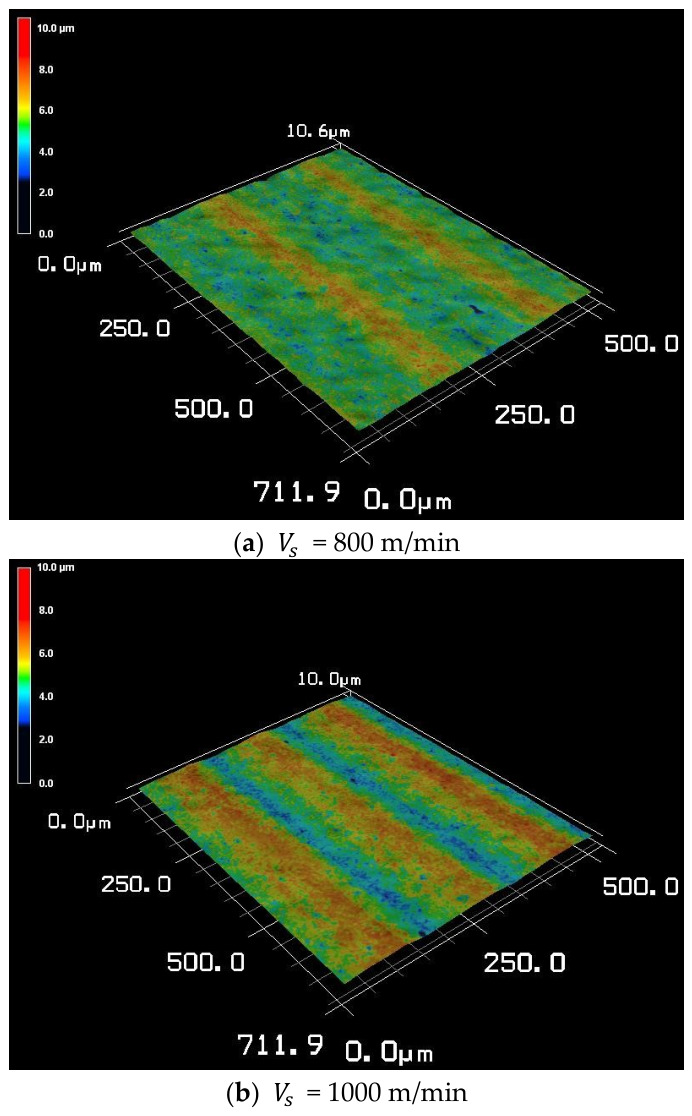

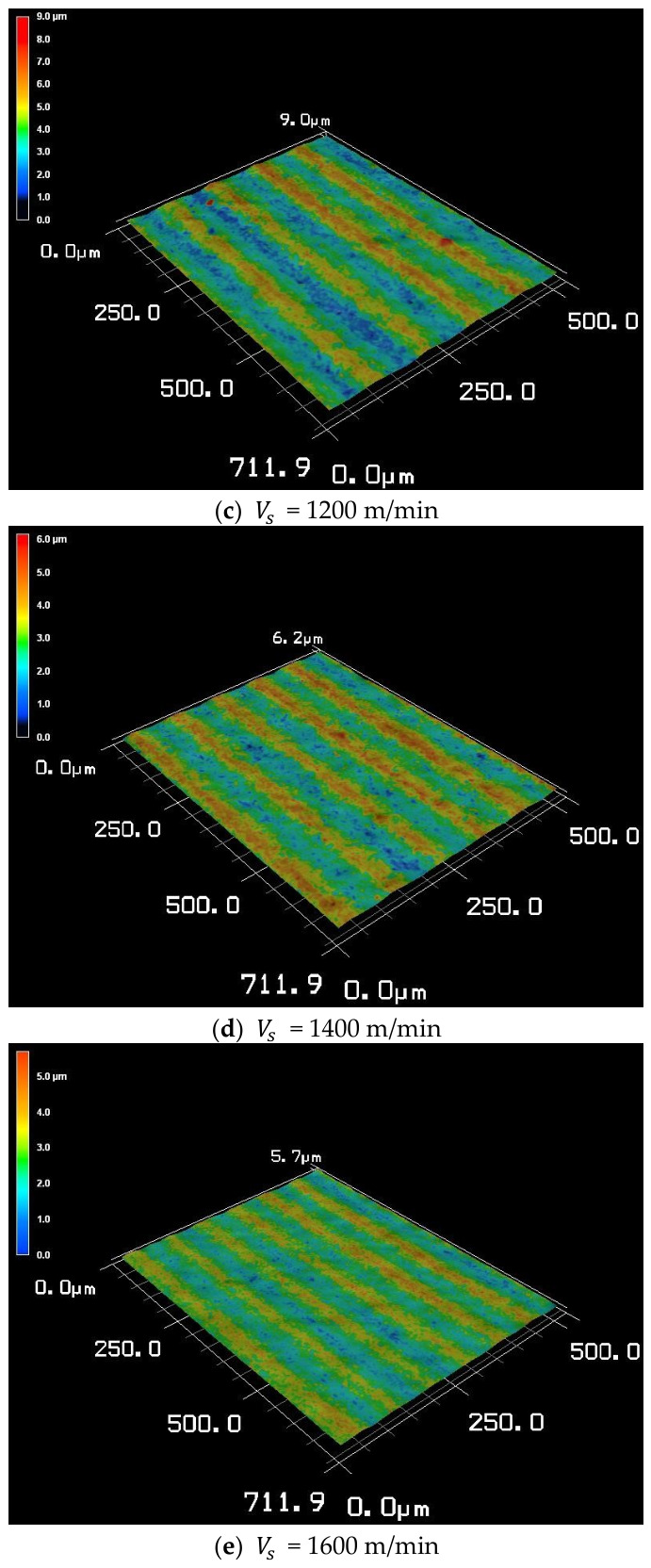
Waviness PV morphology of as-sawn sapphire surface at various wire speeds (application position = 30 mm, $V_{f}$ = 0.3 mm/min, amplitude = 14 µm). (**a**) $V_{s}$ = 800 m/min. (**b**) $V_{s}$ = 1000 m/min. (**c**) $V_{s}$ = 1200 m/min. (**d**) $V_{s}$ = 1400 m/min. (**e**) $V_{s}$ = 1600 m/min.

From Figure 5a–e, it is evident that the PV value of the sapphire slices decreases and the surface becomes flatter as the wire speed increases. This improvement is governed by the combined influence of wire speed and ultrasonic assistance, where the ultrasonic parameters were fixed at an amplitude of 14 μm, a feed speed of 0.3 mm·min^−1^, and a horn position of 30 mm on the wire saw. At lower wire speeds, the abrasives remain in the cutting zone for a longer duration, enabling more vibration interactions. Under these conditions, ultrasonic assistance plays a dominant role by exerting a minor grinding effect on the peaks and valleys of the emerging wire marks, effectively trimming their tips. As the wire speed increases, the abrasives leave the cutting area more rapidly, reducing the number of ultrasonic-induced vibrations and thereby diminishing their direct effect. However, the higher wire speed enhances the sliding motion of the diamond wire, and the abrasives interact more smoothly with the workpiece, which improves the cutting ability exerted by the abrasives and facilitates more stable cutting, resulting in shallower PV. Consequently, even though the ultrasonic contribution is less pronounced at higher wire speeds, its residual effect, combined with the mechanical benefits of increased speed, results in further improvement of surface waviness PV.

#### 3.1.3. Influence of Feed Speed on Surface Waviness PV

**Figure 6:**
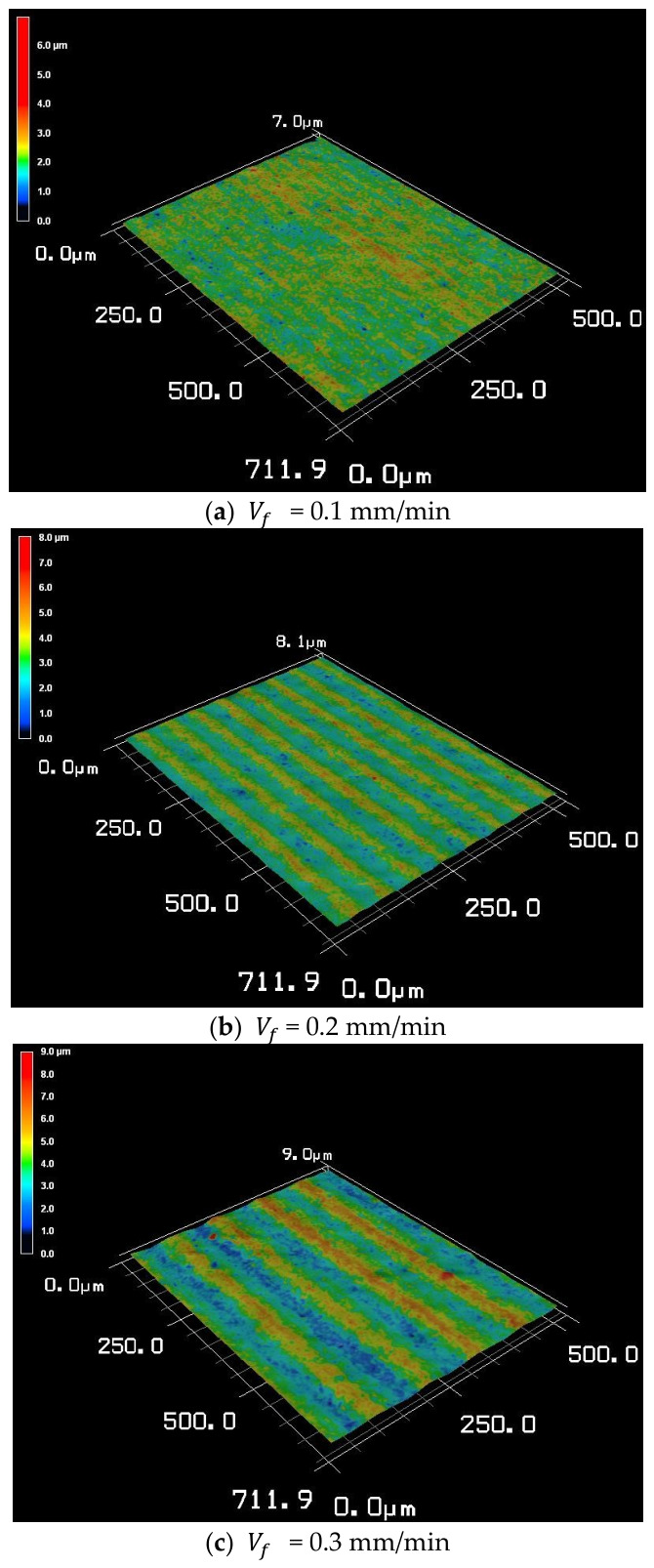

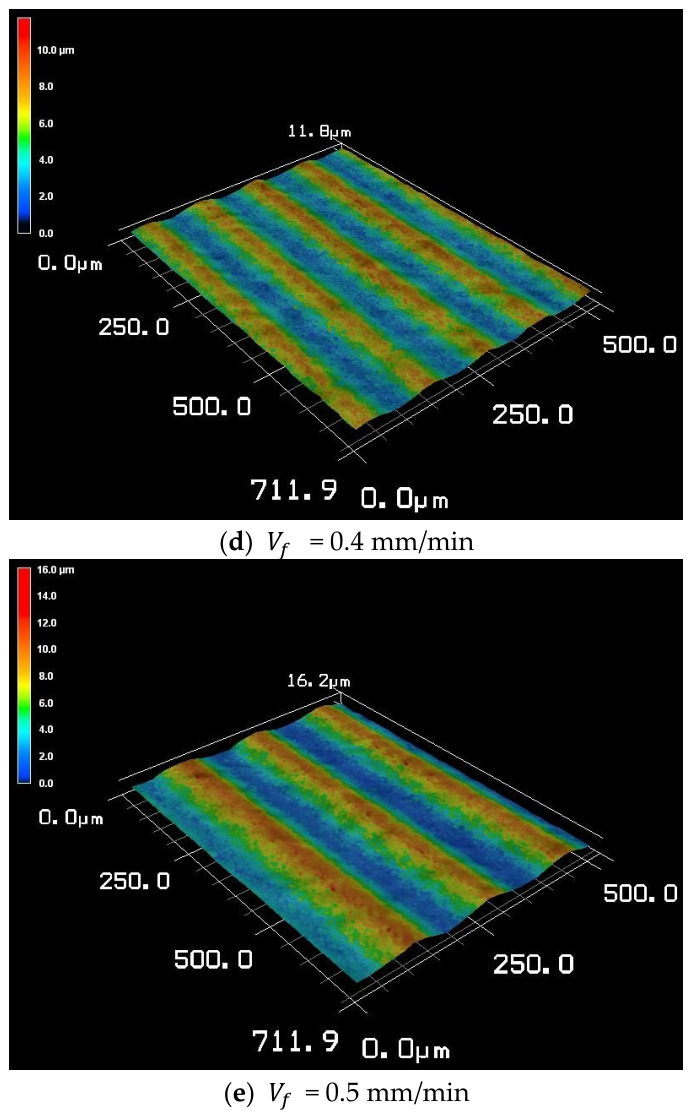
Waviness PV morphology of as-sawn sapphire surface at various feed speeds (application horn position = 30 mm, amplitude = 14 µm, $V_{s}$ = 1200 m/min). (**a**) $V_{f}$ = 0.1 mm/min. (**b**) $V_{f}$ = 0.2 mm/min. (**c**) $V_{f}$ = 0.3 mm/min. (**d**) $V_{f}$ = 0.4 mm/min. (**e**) $V_{f}$ = 0.5 mm/min.

From Figure 6a–e, it is evident that the PV value of the sapphire slices increases and the surface becomes more undulated as the feed speed increases. This behavior is governed by the combined effect of feed speed and ultrasonic assistance, where the ultrasonic parameters were fixed at an amplitude of 14 μm, horn application position 30 mm, and a wire speed of 1200 m·min^−1^. The influence of ultrasonic vibration in this process is closely related to the relative vertical velocity of the abrasives in the cutting zone. As the feed speed increases, the relative vertical abrasive velocity decreases, thereby reducing the intensity of ultrasonic impact on the workpiece surface.

At the lower feed speed of 0.1 mm·min^−1^, ultrasonic vibration plays a dominant role by exerting a minor grinding effect on the peaks and valleys of the emerging wire marks, effectively trimming their tips and reducing surface waviness, as clearly shown in Figure 6a. However, as the feed speed increases, the reduced relative vertical abrasive velocity limits the vibration interactions within the cutting zone, diminishing the direct ultrasonic effect. Meanwhile, higher feed speeds increase cutting resistance and hinder the stable sliding motion of the diamond wire, negatively affecting the cutting ability of the abrasives and leading to higher (PV) values. Nevertheless, even at elevated feed speeds, the residual ultrasonic contribution, although weaker, continues to mitigate excessive surface waviness to some extent.

#### 3.1.4. Influence of Application Horn Position on Surface Waviness PV

**Figure 7:**
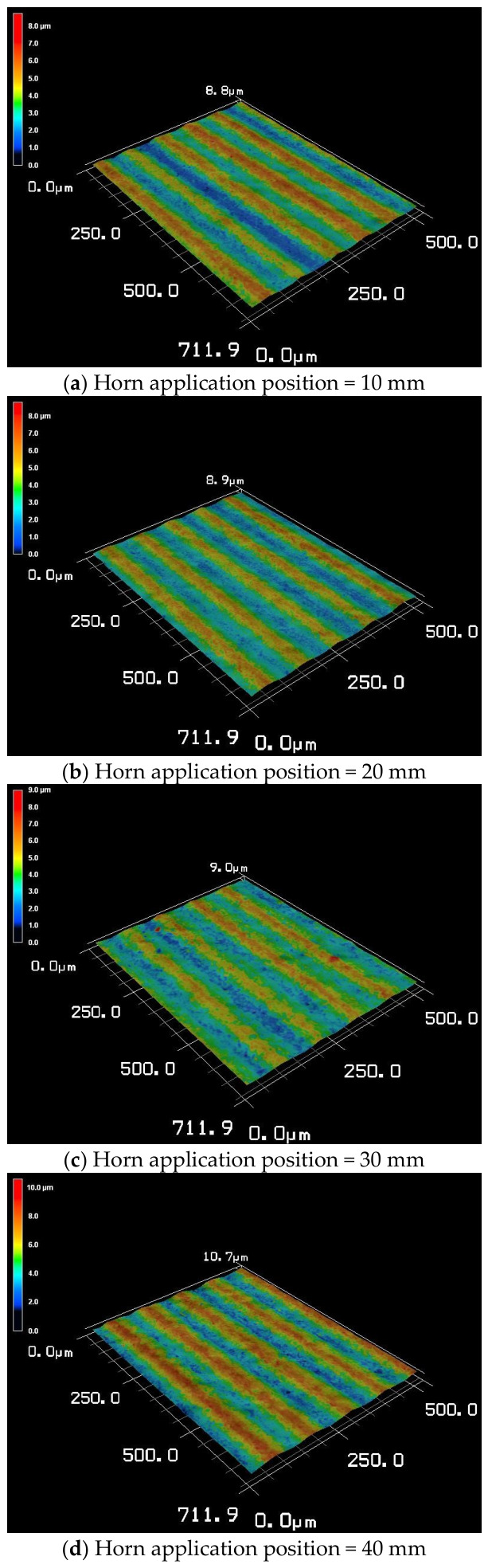

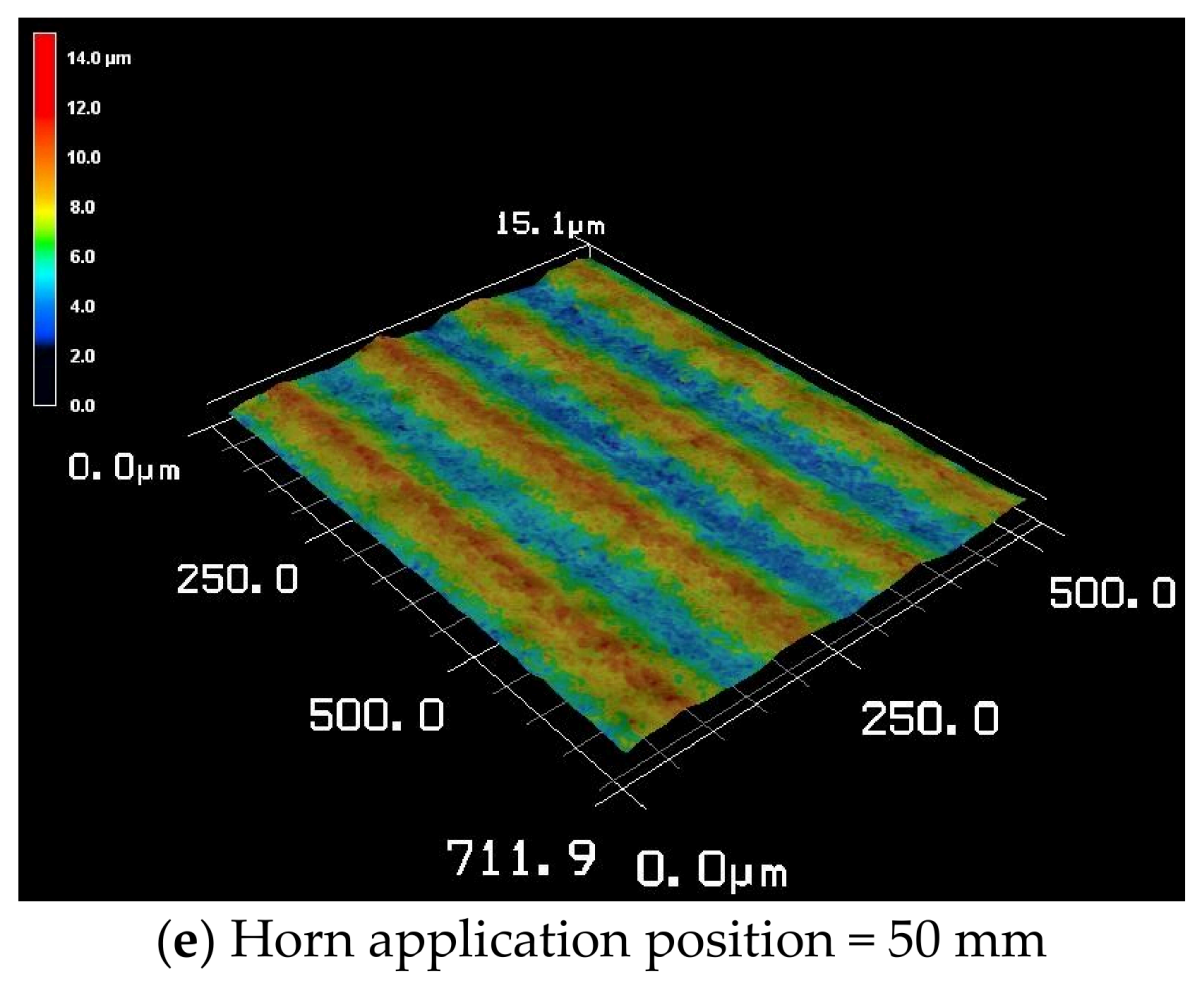
Waviness PV morphology of as-sawn sapphire surface at various horn application positions (amplitude = 14, $V_{f}$ = 0.3 mm/min, $V_{s}$ = 1200 m/min). (**a**) Horn application position = 10 mm. (**b**) Horn application position = 20 mm. (**c**) Horn application position = 30 mm. (**d**) Horn application position = 40 mm. (**e**) Horn application position = 50 mm.

From Figure 7a–e, it is observed that as the application horn position increases from 10 to 50 mm, the PV value of the sapphire slices changes only slightly at 10, 20 mm, and 30 mm, followed by a moderate increase at 40 mm and a more pronounced rise at 50 mm. This indicates that horn position exerts only a minor influence on surface waviness PV within the 10–30 mm range, but the effect becomes more noticeable at larger distances. The ultrasonic parameters were kept constant at an amplitude of 14 μm, a wire speed of 1200 m·min^−1^, and a feed speed of 0.3 mm·min^−1^.

The observed behavior can be attributed to the combined influence of application horn position and ultrasonic energy transmission efficiency. At 10 mm, the horn is located close to the cutting area, enabling efficient energy transfer to the processing zone and maintaining a stable ultrasonic effect. Within the intermediate range of 20–30 mm, energy loss during transmission remains minimal, leading to nearly constant ultrasonic intensity and thus stable PV values. However, when the horn position increases to 40 mm and especially 50 mm, this further leads to more significant energy attenuation along the transmission path and a reduction in the ultrasonic effect, which is reflected by the increased PV, as the diminished micro-hammering weakens the localized smoothing of peaks and valleys formed by wire marks. Overall, these results suggest that horn positions closer to the cutting zone (10–30 mm) are more favorable for ultrasonic vibration to maintain its micro-hammering and minor grinding effect on the peaks and valleys of the emerging wire marks with relatively consistent performance, while greater distances (40–50 mm) introduce noticeable energy losses that increase surface waviness.

### 3.2. Orthogonal Experiment Result Analysis

**Figure 8:**
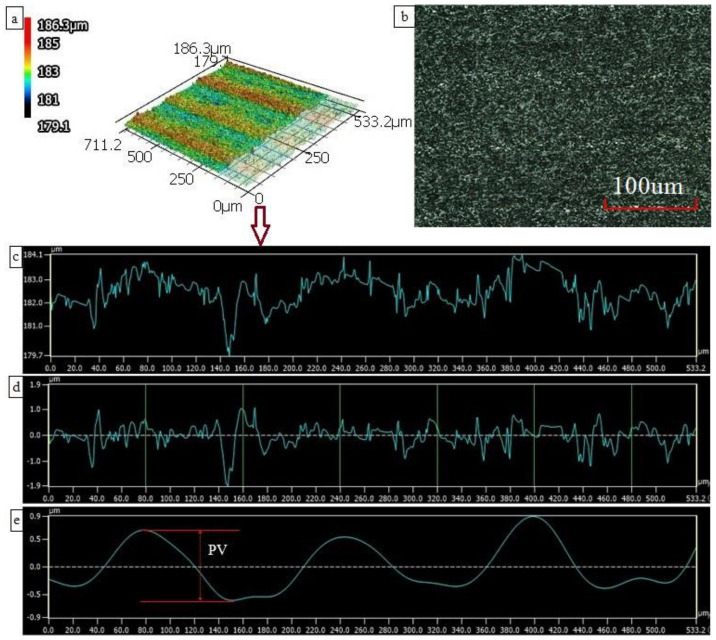
(**a**) 3D surface morphology, (**b**) 2D surface morphology, (**c**) cross-section curve, (**d**) surface roughness *Ra* curve, (**e**) waviness PV curve. The dashed line indicates the zero-height reference line.

After completing the orthogonal experiments, the results were statistically analyzed. The surface roughness (Ra) and waviness (PV) were used as evaluation indices to investigate further the influence of each factor on the experimental results. Figure 8 represents the 2D and 3D surface morphology, along with the three measured curves.

A summary of the experimental data is presented in Table 5.

#### 3.2.1. Degree of Influence of Cutting Parameters on Surface Roughness

A range analysis of the experimental data was conducted to evaluate the extent of influence of each factor, as shown in Table 6. The mean value (K) reflects the degree to which the four experimental factors influence the surface roughness (*Ra*), whereas the range value (R) demonstrates the effect of variations in the levels of each factor on the experimental results.

**Figure 9:**
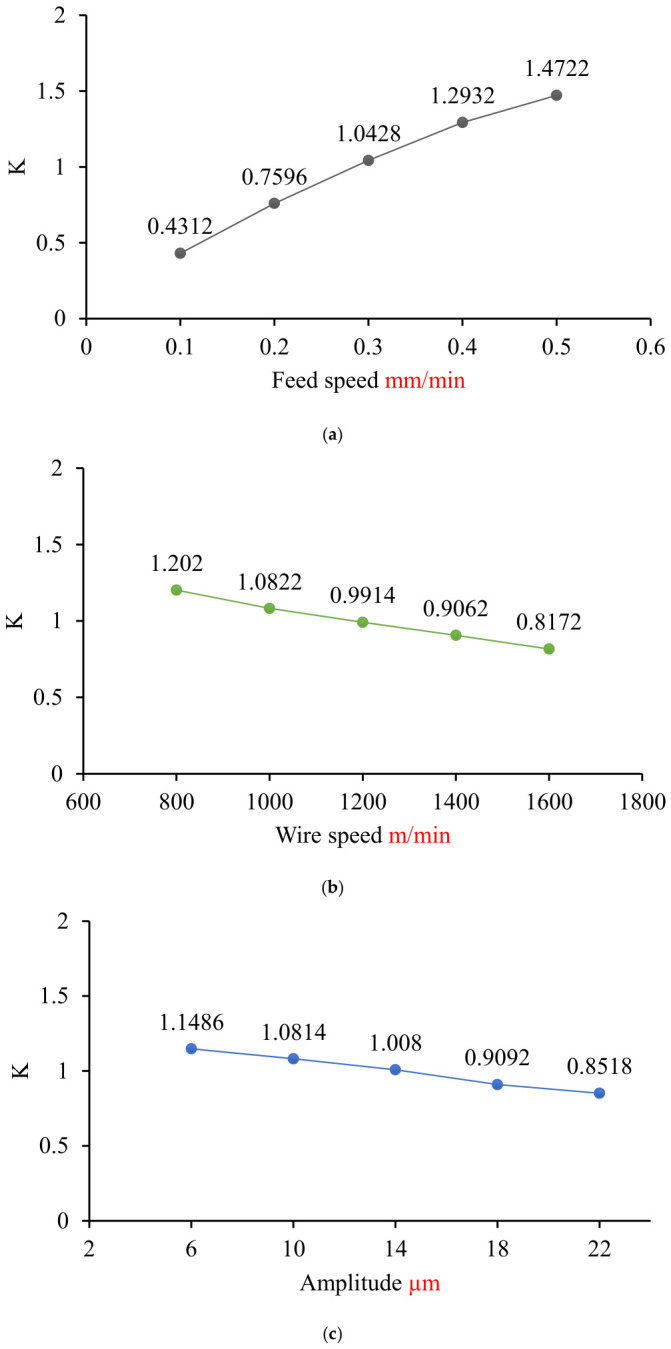

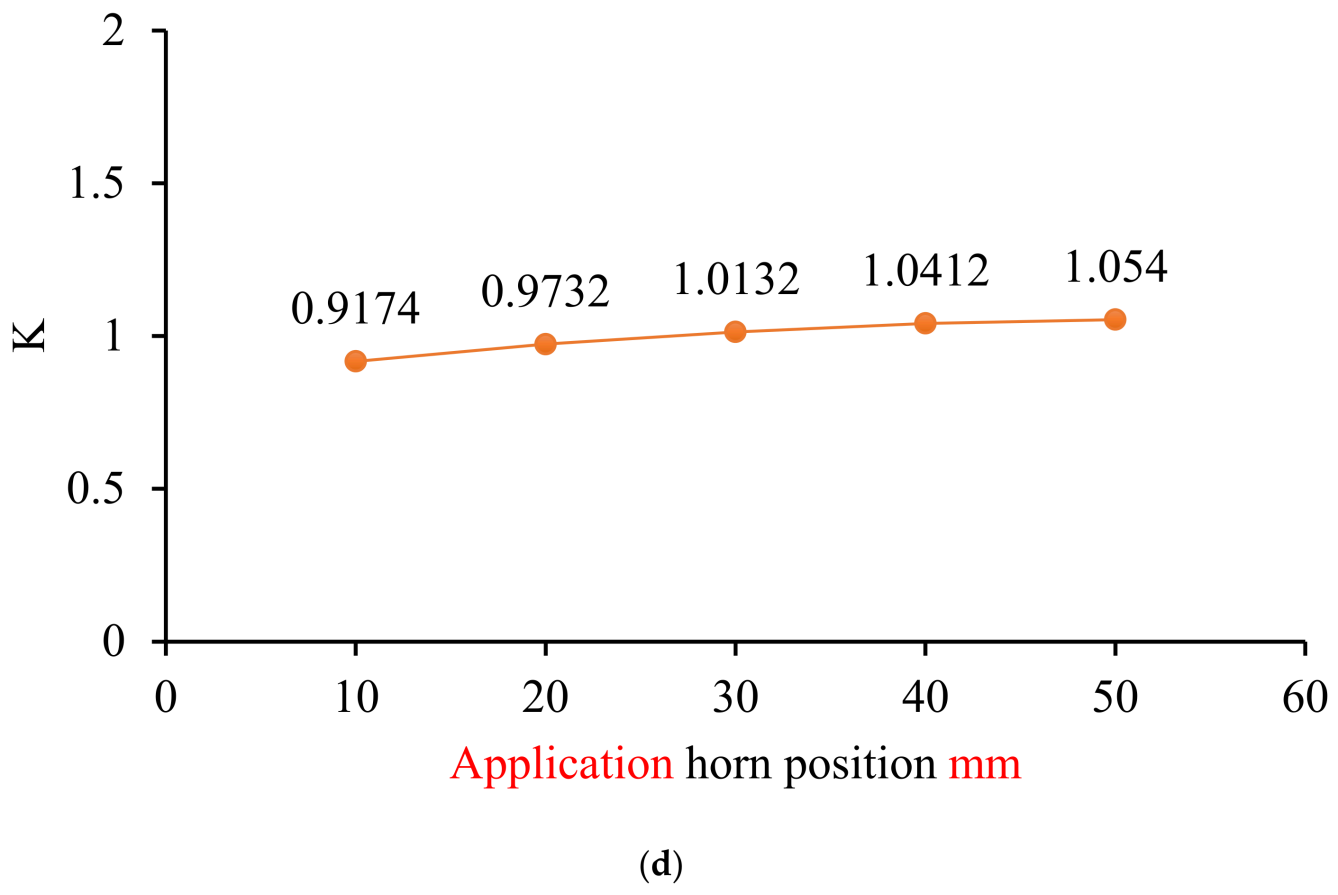
Average of factors affecting Ra at different levels, (**a**) feed speed, (**b**) wire speed, (**c**) amplitude, and (**d**) application horn position. The colored lines connect the mean values at the corresponding factor levels.

A greater range value indicates a more significant impact of that factor’s variation on the experimental outcomes. The data indicate that the R values for workpiece feed rate, diamond wire speed, amplitude, and horn application position are 1.041, 0.3848, 0.2968, and 0.1366, respectively. This indicates that the feed rate exerts the most substantial influence on surface roughness, while the horn application position appears to have the minimal impact. The hierarchy of the sawing parameters’ impact on surface roughness is delineated as follows: feed rate $\left(V_{f}\right)$ takes precedence, followed by wire speed $\left(V_{s}\right)$, then amplitude (*A*), and finally horn application position (*L*). The data presented in Figure 9 indicates a positive correlation between feed rate and horn application position with surface roughness, whereas amplitude and wire speed exhibit a negative correlation. In the specified level ranges of the experimental factors, the most effective combination of processing parameters and ultrasonic parameters is identified as follows: a feed speed of 0.1 mm/min, a wire speed of 1600 m/min, an ultrasonic amplitude of 22 μm, and within the investigated factor levels, L = 10 mm was identified as the best tested horn position. which leads to reduced surface roughness.

Table 7 presents the findings from the variance analysis (ANOVA) concerning surface roughness in the orthogonal experiment. The ranking of F-values indicates that the impact of the sawing parameters on surface roughness diminishes in the following order: feed speed $\left(V_{f}\right)$, wire speed $\left(V_{s}\right)$, amplitude (A), and horn application position (L). This observation is consistent with the results obtained from the range analysis. Additionally, the *p*-values associated with feed speed, wire speed, and amplitude are all below 0.05, suggesting that their influence on surface roughness is statistically significant. The data presented in Figure 9 indicates that the surface roughness of sapphire slices exhibits an increase corresponding to the greater positions of the ultrasonic horn application. Nevertheless, the results from the ANOVA demonstrated that this effect did not reach statistical significance (*p* > 0.05). Although the horn application position L was not statistically significant within the investigated range, it was retained in the *Ra* regression model because of its physical relevance to ultrasonic energy transmission and its modest contribution to predictive performance. When L was excluded, the coefficient of determination decreased from R^2^ = 0.9773 to R^2^ = 0.9562. Therefore, the influence of L on *Ra* was considered minor within the tested range, while retaining it improved the completeness of the predictive model. The observed phenomenon can be attributed to experimental constraints, particularly the safety distance between the ultrasonic application horn position and the workpiece, which cannot be reduced below 10 mm, while distances greater than 50 mm are restricted by machine tool structural limitations. Consequently, the ultrasonic application horn position was confined to a narrow range of 10–50 mm. Within this range, the variation in ultrasonic transmission distance led to minimal energy loss, resulting in insufficient differences between application horn position levels to produce significant changes in *Ra*.

A power regression analysis was performed to determine the relationship between the sawing parameters and *Ra*, utilizing the orthogonal experimental parameters and results. A regression prediction mathematical model for *Ra* was subsequently developed using MATLAB Version 2020 (Natick, MA, USA). The negative indices of $V_{s}$ and A, contrasted with the positive indices of $V_{f}$ and L, suggest that an increase in the speed and amplitude of the wire saw leads to a reduction in the sawed surface roughness. Conversely, an increase in feed speed and shifting position results in an increase in surface roughness.

(1) $$Ra=e^{6.05297}\times V_{f}^{0.78425}\times V_{s}^{-0.6486}\times A^{-0.28164}\times L^{0.05819}$$

#### 3.2.2. Degree Influence of Cutting Parameters on Surface PV

A range analysis of the experimental data was conducted to evaluate the extent of influence of each factor, as shown in Table 8. The mean value (K) reflects the degree to which the four experimental factors influence the surface waviness (PV), whereas the range value (R) demonstrates the effect of variations in the levels of each factor on the experimental results.

**Figure 10:**
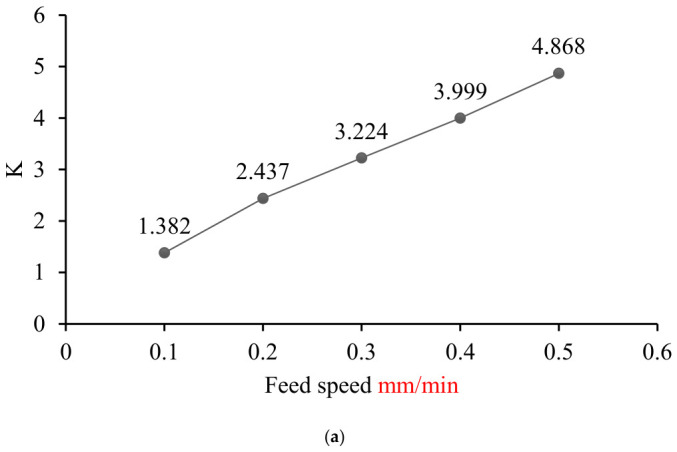

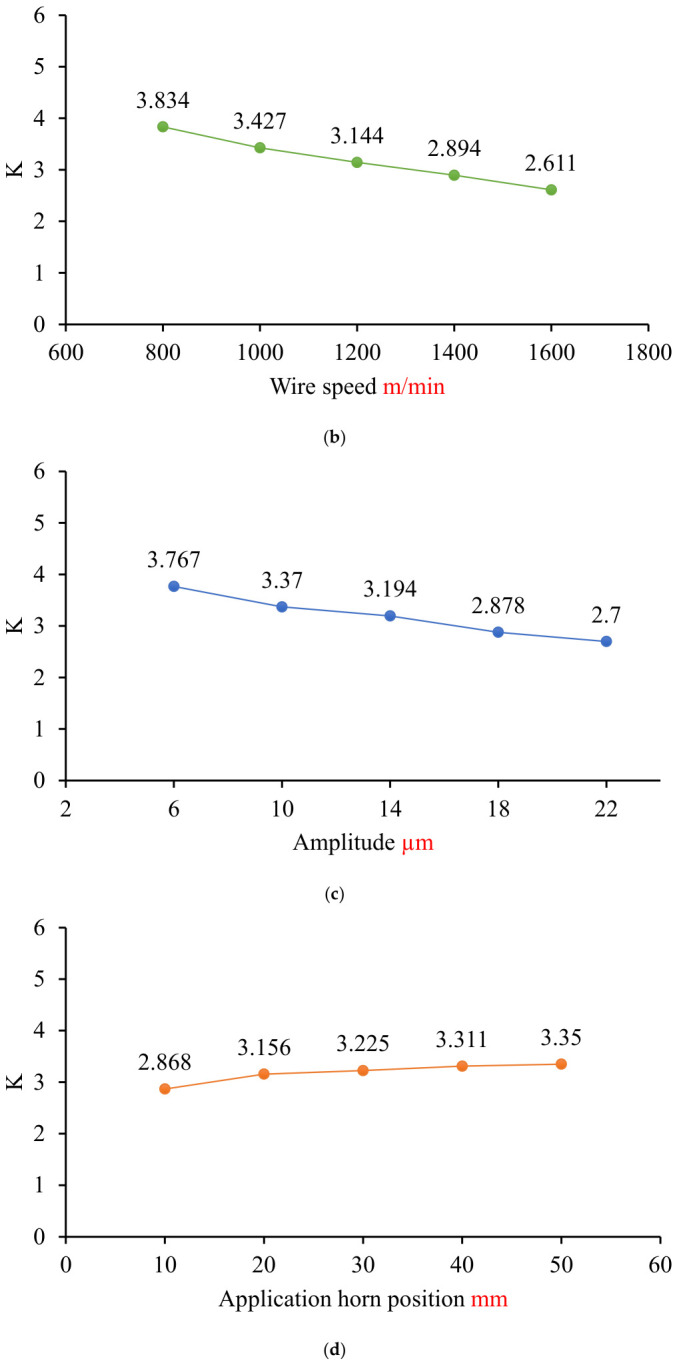
Average of factors affecting PV at different levels, (**a**) feed speed, (**b**) wire speed, (**c**) amplitude, and (**d**) application horn position. The colored lines connect the mean values at the corresponding factor levels.

A greater range value indicates a more significant impact of that factor’s variation on the experimental outcomes. The data indicate that the R values for workpiece feed speed, diamond wire speed, amplitude, and horn position are 3.486, 1.223, 1.067, and 0.481, respectively. This indicates that the feed rate exerts the most substantial influence on surface waviness PV, while the horn application position appears to have the minimal impact. The hierarchy of the sawing parameters’ impact on surface waviness PV is delineated as follows: feed speed ($V_{f}$) takes precedence, followed by wire speed ($V_{s}$), then amplitude (A), and finally application horn position (L). The data presented in Figure 10 indicates a positive correlation between feed rate and application horn position with surface waviness PV, whereas amplitude and wire speed exhibit a negative correlation. In the specified level ranges of the experimental factors, the most effective combination of processing parameters and ultrasonic parameters is identified as follows: a feed speed of 0.1 mm/min, a wire speed of 1600 m/min, an ultrasonic amplitude of 22 μm, and within the investigated factor levels, L = 10 mm was identified as the best tested horn position. which leads to reduced surface waviness PV. These findings are consistent with the Ra range analysis results, reinforcing the accuracy of the experimental assessment.

Table 9 presents the findings from the variance analysis (ANOVA) concerning surface waviness PV in the orthogonal experiment. The ranking of F-values indicates that the impact of the sawing parameters on surface waviness diminishes in the following order: feed speed ($V_{f}$), wire speed ($V_{s}$), and amplitude, horn position (L). This observation is consistent with the results obtained from the range analysis. Additionally, the *p*-values associated with feed speed, wire speed, and amplitude are all below 0.05, suggesting that their influence on surface waviness is statistically significant. The data presented in Figure 10 indicates that the surface waviness PV of sapphire slices exhibits an increase corresponding to the greater positions of ultrasonic application. Nevertheless, the results from the ANOVA demonstrated that this effect did not reach statistical significance (*p* > 0.05). Similarly, although the horn application position L was not statistically significant for PV within the investigated range, it was retained in the PV regression model because of its physical relevance to ultrasonic energy transmission and its modest contribution to predictive performance. Excluding L reduced the coefficient of determination from R^2^ = 0.9740 to R^2^ = 0.9512. Thus, the effect of L on PV was regarded as minor within the tested range, although its inclusion slightly improved model performance. These ANOVA findings are in agreement with those for *Ra*, reflecting the accuracy and reproducibility of the analysis. The observed phenomenon can be attributed to experimental constraints, particularly the safety distance between the ultrasonic horn and the workpiece, which cannot be reduced below 10 mm, while distances greater than 50 mm are restricted by machine tool structural limitations. Consequently, the ultrasonic application position was confined to a narrow range of 10–50 mm. Within this range, the variation in ultrasonic transmission distance led to minimal energy loss, resulting in insufficient differences between position levels to produce significant changes in PV.

A power regression analysis was performed to determine the relationship between the sawing parameters and PV, utilizing the orthogonal experimental parameters and results. A regression prediction mathematical model for PV was subsequently developed using MATLAB Version 2020 (Natick, MA, USA). The negative indices of $V_{s}$ and A, contrasted with the positive indices of $V_{f}$ and L, suggest that an increase in the speed and amplitude of the wire saw leads to a reduction in surface waviness PV. Conversely, an increase in feed speed and shifting position results in an increase in PV. These regression results correspond closely with those obtained for Ra, further confirming the consistency and accuracy of the analysis.

(2) $$PV=e^{7.27423}\times V_{f}^{0.78583}\times V_{s}^{-0.64498}\times A^{-0.31837}\times L^{0.05922}$$

It should be noted that a conventional diamond wire sawing condition without ultrasonic vibration (A = 0) was not included in the present experimental matrix. Therefore, the improvement attributable exclusively to ultrasonic assistance cannot be quantified through a direct paired comparison using the present dataset. Previously reported conventional DWS results for sapphire under comparable wire-speed and feed-speed conditions were considered only as a literature-based benchmark and were not treated as a direct experimental baseline. Accordingly, the present results primarily describe the effects of the investigated UADWS parameters within the ultrasonic amplitude range of 6–22 μm.

## 4. Conclusions

Ultrasonic-vibration-assisted diamond wire sawing (UADWS) experiments on sapphire were conducted to investigate the influence of sawing process parameters and ultrasonic parameters on surface morphology, roughness, and waviness (PV). Both single-factor and orthogonal experiments were performed, and regression models were established from the experimental data. The main conclusions are as follows:

(1) The single-factor experiments reveal that as the amplitude of ultrasonic vibration increases, the application horn position approaches the cutting zone, the wire speed increases, the feed speed decreases, and the waviness PV value of slices decreases.
(2) Range analysis of the orthogonal experiment results indicates that the influence of factors on *Ra* and PV decreases in the order: feed speed > wire speed > ultrasonic amplitude > horn position. The optimal parameter combination is a feed speed of 0.1 mm/min, a wire speed of 1600 m/min, an ultrasonic horn position of 10 mm, and an ultrasonic amplitude of 22 μm.
(3) Regression models for *Ra* and PV were derived as follows:

$$Ra=e^{6.05297}\times V_{f}^{0.78425}\times V_{s}^{-0.6486}\times A^{-0.28164}\times L^{0.05819}$$

$$PV=e^{7.27423}\times V_{f}^{0.78583}\times V_{s}^{-0.64498}\times A^{-0.31837}\times L^{0.05922}$$

Overall, the single-factor experiments clarified the individual effects of ultrasonic amplitude, wire speed, feed speed, and horn application position on the surface waviness PV of sapphire slices, whereas the orthogonal experiments quantified their relative effects on both Ra and PV. The resulting regression models provide a quantitative basis for predicting surface quality and selecting UADWS parameters within the investigated ranges.

This study has several limitations. A conventional DWS condition without ultrasonic vibration (A = 0) was not included in the experimental design; consequently, the improvement attributable exclusively to ultrasonic assistance cannot be quantified directly from the present dataset. In addition, the horn application position was restricted to 10–50 mm because of the required safety distance between the horn and the workpiece and the structural limitations of the machine tool, while the ultrasonic frequency was maintained at a constant value. Future work should conduct paired DWS and UADWS experiments under otherwise identical conditions and investigate broader horn-position and ultrasonic-frequency ranges to validate and extend the present findings.

## Figures and Tables

**Table 1:** Workpiece and diamond wire parameters.

Content	Parameter
Workpiece size (mm)	10 × 10
Length of diamond saw wire (m)	100
Maximum saw wire envelope outer diameter (µm)	220
Fixed abrasives	Nickel-coated diamond
Fixed Abrasive size (µm)	25–35
Fixed Abrasive distribution density (grits/mm)	70–85
Ultrasonic frequency (kHz)	23
Ultrasonic amplitude (µm)	0–23

**Table 2:** Single-factor experiment design table.

Parameters	Amplitude A (µm)	$V_{s}\;(m/min)$	$V_{f}\;(mm/min)$	Position L (mm)
Exp 1	6, 10, 14, 18, 22	1200	0.3	30
Exp 2	14	800, 1000, 1200, 1400, 1600	0.3	30
Exp 3	14	1200	0.1, 0.2, 0.3, 0.4, 0.5	30
Exp 4	14	1200	0.3	10, 20, 30, 40, 50

**Table 3:** Orthogonal experiments: factors and levels.

		Factors		
Levels	Amplitude (µm)	(B) $Position\;L\left(mm\right)$	(C) $V_{s}\;\left(m/min\right)$	(D) $V_{f}\;\left(mm/min\right)$
1	6 $\left(A_{1}\right)$	10 $\left(B_{1}\right)$	800 $\left(C_{1}\right)$	0.1 $\left(D_{1}\right)$
2	10 $\left(A_{2}\right)$	20 $\left(B_{2}\right)$	1000 $\left(C_{2}\right)$	0.2 $\left(D_{2}\right)$
3	14 $\left(A_{3}\right)$	30 $\left(B_{3}\right)$	1200 $\left(C_{3}\right)$	0.3 $\left(D_{3}\right)$
4	18 $\left(A_{4}\right)$	40 $\left(B_{4}\right)$	1400 $\left(C_{4}\right)$	0.4 $\left(D_{4}\right)$
5	22 $\left(A_{5}\right)$	50 $\left(B_{5}\right)$	1600 $\left(C_{5}\right)$	0.5 $\left(D_{5}\right)$

**Table 4:** Experimental parameter combinations.

No.	Combinations				No.	Combinations			
1	A1	B1	C1	D1	14	A3	B4	C1	D3
2	A1	B2	C2	D2	15	A3	B5	C2	D4
3	A1	B3	C3	D3	16	A4	B1	C4	D2
4	A1	B4	C4	D4	17	A4	B2	C5	D3
5	A1	B5	C5	D5	18	A4	B3	C1	D4
6	A2	B1	C2	D3	19	A4	B4	C2	D5
7	A2	B2	C3	D4	20	A4	B5	C3	D1
8	A2	B3	C4	D5	21	A5	B1	C5	D4
9	A2	B4	C5	D1	22	A5	B2	C1	D5
10	A2	B5	C1	D2	23	A5	B3	C2	D1
11	A3	B1	C3	D5	24	A5	B4	C3	D2
12	A3	B2	C4	D1	25	A5	B5	C4	D3
13	A3	B3	C5	D2					

**Table 5:** Results of the orthogonal experiment.

No.	*Ra* (µm)	PV (µm)
1	0.621	2.088
2	0.940	3.175
3	1.248	4.020
4	1.405	4.414
5	1.529	5.140
6	1.154	3.410
7	1.326	4.041
8	1.437	4.700
9	0.381	1.300
10	1.109	3.400
11	1.359	4.450
12	0.345	1.114
13	0.578	1.815
14	1.323	4.039
15	1.435	4.550
16	0.555	1.793
17	0.700	2.200
18	1.402	4.391
19	1.481	4.800
20	0.408	1.208
21	0.898	2.600
22	1.555	5.250
23	0.401	1.200
24	0.616	2.000
25	0.789	2.450

**Table 6:** Range analysis of *Ra*.

Item		Factors		
	A (µm)	L (mm)	$V_{s}$ (m/min)	$V_{f}$ (mm/min)
K_1_	5.743	4.587	6.01	2.156
K_2_	5.407	4.866	5.411	3.798
K_3_	1.008	5.066	4.957	5.214
K_4_	0.9092	5.206	4.531	6.466
K_5_	0.8518	5.270	4.086	7.361
${\bar{K}}_{1}$	1.1486	0.9174	1.2020	0.4312
${\bar{K}}_{2}$	1.0814	0.9732	1.0822	0.7596
${\bar{K}}_{3}$	1.0080	1.0132	0.9914	1.0428
${\bar{K}}_{4}$	0.9092	1.0412	0.9062	1.2932
${\bar{K}}_{5}$	0.8518	1.0540	0.8172	1.4722
R	0.2968	0.1366	0.3848	1.0410

**Table 7:** Analysis of variance for *Ra*.

Source	Degree of Freedom	Adj SS	Mean Square	F-Value	*p*-Value
A	4	0.29490	0.073725	18.12	0.000
L	4	0.06164	0.015411	3.79	0.052
$V_{s}$	4	0.44924	0.112311	27.60	0.000
$V_{f}$	4	3.46048	0.865120	212.62	0.000
Error	8	0.03255	0.004069		
Total	24 R^2^ = 0.9773	4.29882			

**Table 8:** Range analysis results of PV.

Item		Factors		
	A (µm)	L (mm)	$V_{s}$ (m/min)	$V_{f}$ (mm/min)
$K_{1}$	18.837	14.341	19.168	6.91
$K_{2}$	16.85	15.78	17.135	12.183
$K_{3}$	3.194	16.126	15.719	16.12
$K_{4}$	14.392	16.553	14.471	19.996
$K_{5}$	13.5	16.748	13.055	24.34
${\bar{K}}_{1}$	3.767	2.868	3.834	1.382
${\bar{K}}_{2}$	3.370	3.156	3.427	2.437
${\bar{K}}_{3}$	3.194	3.225	3.144	3.224
${\bar{K}}_{4}$	2.878	3.311	2.894	3.999
${\bar{K}}_{5}$	2.700	3.350	2.611	4.868
R	1.067	0.481	1.223	3.486

**Table 9:** Analysis of Variance for PV.

Source	Degrees of F	Adj SS	Mean Square	F-Value	*p*-Value
A	4	3.5137	0.87843	18.28	˂0.001
L	4	0.7282	0.18205	3.79	0.052
$V_{s}$	4	4.4747	1.11867	23.28	˂0.001
$V_{f}$	4	36.5389	9.13473	190.11	˂0.001
Error	8	0.3844	0.04805		
Total	24 R^2^ = 0.974	45.6399			

## Data Availability

All data generated or analyzed during this study are included in this published article.
